# Clinicopathological features and androgen receptor expression in triple negative breast cancer at Lagos, Nigeria

**DOI:** 10.3332/ecancer.2022.1452

**Published:** 2022-10-03

**Authors:** Muibat Haruna, Adetola Olubunmi Daramola, Nicholas Awodele Awolola, Nahla Mustafa Badr, Adekunbiola Aina Fehintola Banjo, Abeer Shaaban

**Affiliations:** 1 Lagos University Teaching Hospital, Lagos 100254, Nigeria; 2 Department of Anatomic and Molecular Pathology, College of Medicine, University of Lagos, Lagos 101014, Nigeria; 3 Department of Pathology, Faculty of Medicine, Menoufia University, Shebin Elkom 32511, Egypt; 4 Institute of Cancer Science and Genomics, University of Birmingham, Birmingham B15 2WB, UK; 5 Queen Elizabeth Hospital Birmingham and University of Birmingham, Birmingham B15 2WB, UK

**Keywords:** triple negative breast cancer, quadruple negative breast cancer, androgen receptor

## Abstract

**Introduction:**

Androgen receptor (AR) is one of the predominant nuclear hormone receptors in invasive breast cancer and can be explored as a biomarker of response for targeted anti-androgen therapy, especially in the setting of triple negative breast cancer (TNBC). Luminal AR is a distinct subtype amongst TNBC cases following gene expression studies. TNBC is higher in Africans (23%–82%) and African-Americans (29.8%) compared to Caucasian (10%–15%) breast cancer patients; however, there is a paucity of data on AR expression in this population. The aim of this study is to determine the expression of AR and the proportion of AR positive cancers in TNBCs at the Lagos University Teaching Hospital, Lagos, Nigeria.

**Methodology:**

Out of 99 reviewed cases, 78 formalin fixed, paraffin embedded TNBC cases were assembled into a tissue microarray, stained and analysed for AR expression using immunohistochemistry.

**Results:**

The mean age of the TNBC patients was 49.3 years (range: 20–80 years). The histologic types in this study were invasive carcinoma (no special type) 75.4%; metaplastic carcinoma 21.4%; lobular carcinoma and mucinous carcinoma 1.6% each. Of 61 TNBC cases analysed, 37.7% were AR positive and 62.3% were AR negative, making the latter to become quadruple negative breast cancers. There was a significant association between age and AR expression (*p* = 0.02). In the subjects that expressed AR positivity, patients below 50 years accounted for 34.8% (8 of 23) while 65.2% (15 of 23) were above 50 years. There was no significant association between AR expression and histologic type or tumour grade.

**Conclusion:**

Over a third of this Nigerian TNBC cohort study is AR+. This warrants further exploration of the predictive and prognostic significance of its expression amongst TNBC and the potential for targeted therapy, specifically androgen antagonists to improve the outcome of this disease with limited therapeutic options.

## Introduction

Breast cancer has been identified as a heterogeneous disease with marked differences in morphology, molecular biology, clinical findings and outcome [1, 2]. Triple negative breast cancer (TNBC) is defined by the absence of expression for oestrogen receptor (ER), progesterone receptor (PR) and human epidermal growth factor receptor-2 (HER2) which has limited targeted therapeutic options. TNBC cases are generally treated by surgery, chemotherapy and carry a poor prognosis when compared with luminal breast cancer.

TNBC accounts for 170,000 cases of the global breast cancer burden and has been associated with disparities in racial incidence [3]. It accounts for 10%–15% of all breast cancer cases, with a much higher incidence in women of African ancestry [4]. The frequency of TNBC is two to three times higher in African-Americans when compared to Caucasians studies with studies in Africa ranging between 23% and 82%, and higher frequencies been reported in Western sub-Saharan Africa [5–7]. It is also associated with young age of presentation, advanced stage at diagnosis, high mitotic index and breast cancer gene 1 (BRCA1) mutation in African, African-American and Hispanic women [8, 9].

A large Nigerian data series, from the South-West geo-political zone, highlighted that breast cancer comprised a large proportion of TNBC. TNBC was the predominant molecular phenotype accounting for 47.65% of 835 tumours analysed, followed by the HER2 positive subtype (19.6%) [10]. At the Lagos University Teaching Hospital (LUTH), in 2016, it was reported that TNBC accounted for 35% of their cohort of 115 breast cancer cases. Over half (52.5%) of these TNBC subjects had a basal phenotype [11]. Another hospital-based study, at the Jos University Teaching Hospital in North-Central Nigeria, demonstrated a prevalence of 41.3% (26 cases) out of the 63 cases [12]. Liman *et al* [13] and Usman *et al* [14] reported 13.48%(89 of 660) and 46.6% (55 of 118) TNBC cases in North-Western Nigeria, respectively. In North-Eastern Nigeria, Minoza *et al* [15] reported a higher percentage of 52.6% (20 of 38) TNBC cases while Ukah *et al* [16] in South-Eastern Nigeria reported 46.5% (40 of 86) TNBC cases in their study population, respectively. However, there is a paucity of data regarding androgen receptor (AR) expression in TNBC in the high-risk African population.

According to Lehmann *et al* [2], TNBC is categorised into four subcategories based on genetic expression profiles, namely, basal-like 1 and basal-like 2, mesenchymal (M) and luminal AR (LAR). These subcategories of TNBC are noted to have unique ontologies and differential response to therapy [17, 18].

AR, like ER and PR, is a steroid receptor that is expressed in normal breast as well as 60%–90% of all breast cancer [1]. The expression of AR (60%–90%) may exceed that of ER (70%–80%) and PR (50%–70%) [19, 20]. There is a substantial variability in the frequency of AR expression in breast cancer of various subtypes including TNBC, with a wide range of 6.6%–75%. This is primarily due to variation in study designs particularly in the number of patients, definition of molecular subtypes and the threshold cut off used for AR positivity [8, 21–23]. A Ghanaian study of 147 invasive breast cancer cases had majority (61%) reported as TNBC. AR positivity was 44% in the entire study population while 24% of the TNBC are AR positive [5]. A Yale study in the USA reported that of 400 invasive breast cancers cases, 12.5% were TNBC while 8% were quadruple negative breast cancer (QNBC) and 33% of TNBCs were AR positive [24]. QNBC is defined as TNBCs that also lack expression of AR [25–27]. An Italian non-interventional retrospective study of 45 TNBC cases evaluated for the expression of AR, E-cadherin and Ki-67 in relation to histologic type, time of relapse and overall survival (OS) observed an AR positivity of 26.6% amongst TNBC cases. AR and Ki-67 expression were independent predictors for OS in multivariate analysis while AR negativity and Ki-67 index were significantly correlated with poor prognosis [25].

It is of note that AR is uniformly and diffusely expressed in apocrine metaplasia, a component of fibrocystic change that commonly lacks the expression of ER and PR [1, 8, 24]. It is similarly expressed in classic apocrine carcinoma and has recently been highlighted as a possible candidate for targeted therapy especially in the setting of TNBC and cancers with apocrine signature [5, 24]. Androgens, including testosterone and dihydrotestosterone are involved in the normal function of multiple female organs, including the reproductive tract, bones, kidneys and muscles [1, 8]. They act indirectly as pro-hormones of oestradiol or directly by binding to AR. The binding of circulating androgens to AR leads to translocation of the receptor to the nucleus, binding to target genes and transcription activation [1, 28, 29]. Due to the absence of targeted therapies, surgery and cytotoxic chemotherapy are the mainstay of treatment in TNBC, yet outcomes are poor when compared with other subtypes [9, 17]. The subset of TNBC that expresses AR, typically LAR subtype, may be a target for therapy. Androgen deprivation therapy has long been used to treat orchidectomy resistant prostate cancer, and more recently, LAR cell line has shown a unique sensitivity to bicalutamide (AR antagonist) [17]. These data may be useful in biomarker selection, drug discovery and clinical trial design appropriately targeted to TNBC.

However, the proportion and significance of AR positivity are yet to be determined in Nigerian TNBC. This study aims to analyse the clinic-pathological features and immunohistochemical expression of ARs in TNBC at the LUTH, its prognostic significance and the stratification of TNBC into AR positive and AR negative with the latter to be designated as QNBC.

## Materials and methods

This is a cross sectional retrospective study of female patients with ER/PR/HER2 negative breast cancer diagnosed from 2016 to 2019 in the Department of Anatomic and Molecular Pathology, LUTH, Idi-Araba, Lagos, Nigeria. Ethical approval was sought and granted by the LUTH Health Research & Ethics Committee (HREC no: 2752). ER/PR and/or HER2 positive invasive breast carcinoma; pure ductal carcinoma *in situ* (DCIS)/lobular carcinoma *in situ* and all male breast cancers were excluded from the study.

Ninety-nine cases of formalin fixed paraffin embedded tissue blocks of consecutive invasive breast cancers and immunohistochemistry (IHC) slides for ER/PR and HER2 over the study period were retrieved from the pathology database. The pathology data including patient’s age, grade, staging and pathology report of TNBC cases were made available and extracted from the database. Survival data could not be obtained due to sparse records at the originating hospital. Fresh sections from the selected tissue blocks were cut and stained with haematoxylin and eosin (H&E). Representative H&E sections of all cases were examined by M.H and jointly reviewed by A.M.S (a specialist UK based breast pathologist), to confirm the diagnosis of invasive breast carcinoma, histologic type and histologic grade, presence or absence of lymphovascular invasion and DCIS.

Of the 99 cases reviewed, 14 had to be excluded for the following reasons: one autolysed tissue, two crushed tissue samples, six due to small tumour size, one due to poor tissue processing, one tumour of non-breast origin and lastly three cases with high grade DCIS only. Repeat IHC for ER, PR (using Dako Agilent Autostainer Link for ER and PR) and HER2 (using Ventana BenchMark ULTRA autostainer) was performed as previously described [8]. The repeat IHC further excluded 7 more cases which expressed ER (*n* = 4) and HER 2 (*n* = 3). Only 78 cases were assembled on TMA. All the reviews were done blindly following the Royal College of Pathology guidelines [22].

### Tissue microarray (TMA) construction

Following histological review, tumour areas of interest were marked on the tumour donor blocks and a tissue microarray (TMA) was constructed as previously described by Pinder *et al* [30]. Firstly, a tissue microarray map was constructed which was used to order a TMA block. A manual TMA block was used to assemble two 0.6 mm core punches per case in duplicates. Two cores were punched out from the marked areas, which were from the centre and periphery of the tumour donor blocks and arrayed into the pre-punched hole in the recipient block. From the TMA block, 4 µm sections were cut and sections mounted onto charged slides and dried overnight before staining.

### Immunohistochemistry

Standard IHC was applied on deparaffinised sectioned slides. Antigen retrieval was performed by warm incubation in ethylenediaminetetraacetic acid (EDTA) buffers (pH8). This was performed at 95°C for 10 minutes. The section was incubated for 20 minutes using a ready to use primary antibody AR 441 (mouse monoclonal) in a Dako autostainer along with a batch of positive tissue control. Endogenous enzyme was inactivated or inhibited with peroxidase inhibitor and then washed before antigen retrieval. This was incubated with Rabbit anti-mouse horseradish peroxidase (a conjugated secondary antibody) for 30 minutes at room temperature. The colour was developed using diaminobenzidine as a chromogen. Finally, this was counter-stained with haematoxylin for 15 seconds. Immunostaining was interpreted as positive or negative using a cut off of 1% and using the Quick/Allred score of ≥3 for positivity [24].

### Androgen receptor scoring

In this study, we used the cut-off of ≥1% and semi-quantification for allocation of positivity. American Society of Clinical Oncology/College of American Pathologists (ASCO/CAP) guideline recommends a cut-off point of ≥1% for ER and PR positivity. Therefore, we applied the same threshold of ≥1% proposed by the 2020 ASCO/CAP guideline for AR and used the same quick scoring method. Only nuclear staining was considered, and the entire invasive component was assessed [31]. The percentage or proportion of tumour cells staining positively was scored manually and recorded. The measurement of average intensity of the positively stained tumour cells on the entire section was relative to the intensity of positive controls ran with the same batch.

## Results

### Age distribution of TNBC patients

**Figure 1.**
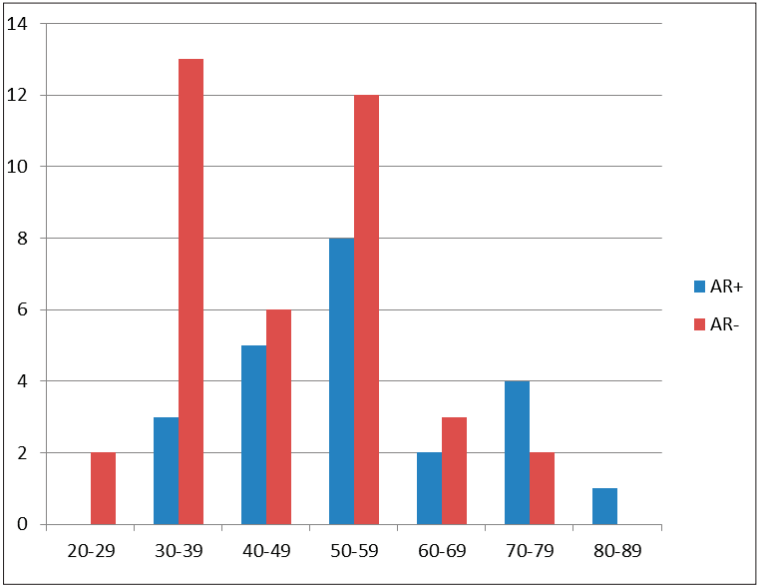
Age (years) distribution in relationship to frequency of AR expression in TNBC showing a bimodal age (years) peak.

Following histological review, ER/PR/HER2 testing and TMA analysis, a total of 61 TNBC cases fulfilled the inclusion criteria for this study. The mean age was 49.3 years (standard deviation ±13.4) with a range of 24–80 years (Figure 1). Of these patients, 47.5% were under the age of 50 while 52.5% were 50 years or older.

### Histological features of the Nigerian TNBC

The predominant histologic types were invasive carcinoma, no special type (NST), 46 of 61 (75.4%) and metaplastic carcinoma 13 of 61 (21.4%). One case each of invasive lobular carcinoma and invasive mucinous carcinoma 1 of 61 (1.6%) was seen (Table 1). While there was no grade 1 invasive breast cancer, there were 29 (47.5%) grade 2 and 32 (52.5%) grade 3 invasive breast cancers. Using the components of histological grading (nuclear pleomorphism, tubule formation, mitoses), most grade 2 cancers had Elston Ellis modification of the Scarff_Bloom Richardson grading system (ESBR) score of 7 (26 of 29) with a minority scoring 6 (3 of 29) [32]. Amongst the grade 3 invasive breast cancers, more than half had ESBR score of 8 (18 of 32) while the rest had ESBR score of 9 (14 of 32).

### Expression of AR in Nigerian TNBC

**Figure 2.**
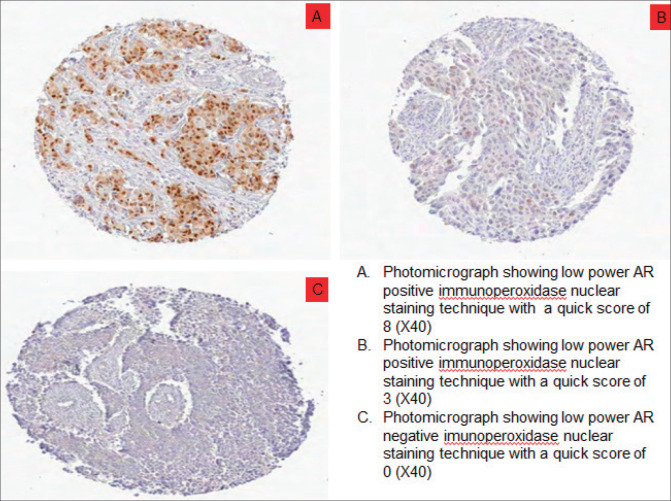
Photomicrographs showing AR expression of TNBCs. (a): Low power AR positive immunoperoxidase nuclear staining technique with a quick score of 8 (×40). (b): Low power AR positive immunoperoxidase nuclear staining technique with a quick score of 3 (×40). (c): Low power AR negative immunoperoxidase nuclear staining technique with a quick score of 0 (×40).

**Figure 3.**
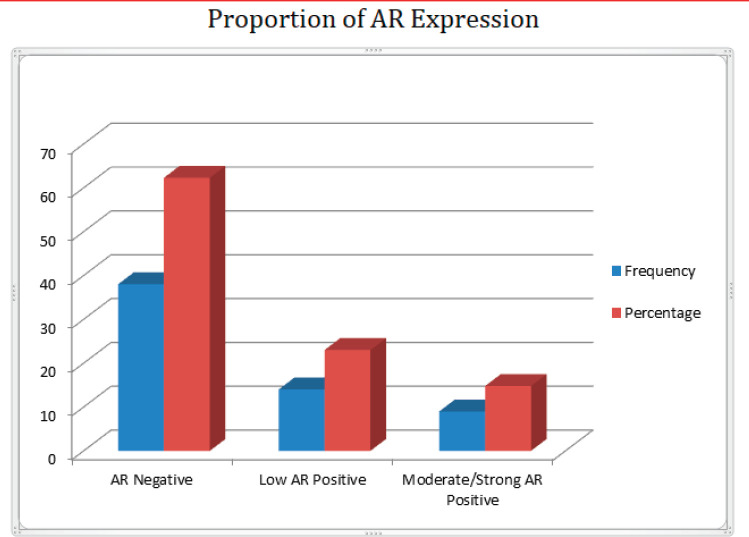
Bar chart showing the frequency and percentage of AR proportion in TNBC.

Out of 61 TNBCs evaluated for AR expression, 23 (37.7%) demonstrated a positive nuclear AR staining and 38 (62.3%) were AR negative (QNBC). Of the AR positive TNBC cases, 14 (23.0%) indicated a low AR positive expression (AR proportion between 1% and 10%) while 9 (14.8%) were moderately or strongly positive for AR expression [31]. This is seen in Figures 2 and 3. There was a significant association between age and AR expression (*p* = 0.02). Two peaks in the age distribution of AR positive TNBC were observed, 30–39 years and 50–59 years with a two-decade gap between them as shown in Figure 1. No AR positivity was seen in TNBC from patients under the age of 30.

### Association of AR expression with tumour characteristics

There was no significant association between the expression of AR in TNBC cases and histologic types. Tumour grade was relatively higher among AR negative subjects compared to AR positive subjects, though this was not statistically significant. The predominant histologic type was invasive carcinoma (NST) in both AR positive and AR negative categories. Within the AR positive tumours, invasive carcinoma NST accounted for 19 of 23 cases (82.6%), metaplastic carcinoma accounted for 3 of 23 cases (13.0%) and invasive mucinous carcinoma for 1 of 23 cases (4.4%). Amongst the TNBC that were AR negative, invasive carcinoma NST accounted for 27of 38 cases (71.1%), metaplastic carcinoma 10 of 38 cases (26.3%) and invasive lobular carcinoma 1of 38 cases (2.6%).

## Discussion

The development of targeted therapy is the ideal goal in cancer management, which had been successful with ER and HER2 positive breast cancers [29]. Studies have shown that only 20%–30% of patients with TNBC achieved complete pathologic response on neoadjuvant chemotherapy and have demonstrated same prognosis as in patients with non-TNBC. However, the groups of TNBCs without complete pathologic response have a higher relapse rate and poor prognosis. The responses of TNBCs to adjuvant chemotherapy have been associated to the different subtypes which can serve as a possible target for individualised treatment [5, 6, 36]. In this study, we aimed to determine the proportion of TNBC that expressed AR for further stratification into AR positive TNBC and QNBC. The expression of AR would allow for individualised treatment based on tumour biology [37]. Studies on African TNBC patients to date have been limited by sample size with the exception of few large-scale studies. This current study evaluated 99 tumours originally diagnosed as TNBC. Thorough histological review and repeat hormone receptor and HER2 testing reclassified a number of these lesions, further highlighting the importance of comprehensive histological review and standardised protocols for IHC staining and scoring. Cases which accounted for 36% of invasive breast cancers seen over a 4-year period from 2016 to 2019 were included. Previous studies have reported higher proportion of TNBC in people of African descent, including African Americans [4–7]. In Nigeria, the frequency of TNBC ranges between 13.48% and 52.6% while in Ghana, TNBC constituted 61% of the 147 cases of invasive breast cancer cases studied by Proctor *et al* [5, 11–16]. In Yale USA, Safarpour *et al* [24] reported 12.5% TNBC out of 400 cases of invasive breast cancer investigated. Of 1,994 cases of primary operable invasive breast carcinoma in Nottingham, United Kingdom, Rakha *et al* [33] reported 16.3% to be of the TNBC phenotype. Mohammadizadeh *et al* [29] studying an Iranian cohort reported a 17.1% frequency out of 70 invasive breast carcinomas analysed. In 2016, Asano *et al* [34] in Japan observed that 34.5% were TNBC out of a total of 177 patients with resectable early stage breast cancer. The ethnic background is therefore likely to influence the frequency of breast cancer molecular subtypes.

The mean age for index study is 49.3years and the mean for Zaria TNBC study population is 42.89 ± 11.88 years which falls under 50 years of age and is similar to median ages of under 50 years reported by Ghanaian (45 years), Egyptian (35.6 years) and Nottingham (49.9 years) study cohorts, respectively [5, 13, 33, 35]. However, the median ages for Yale based study in the USA (65 years), an Italian cohort (58.8 years) and mean age for Iranian population (50.94 years) were all over 50 years [24, 25, 29]. Other studies also corroborate the younger age of incidence as well as the poor prognosis of TNBC in Africans and African-Americans when compared to Caucasians. The mean/median ages for the African and Caucasian studies are highlighted in Table 2.

There was a significant association between age and AR expression in the current study. In the subjects with AR positive tumours, most patients (65.2%) were over 50 years of age. This expression in the older patients has also been described in a US TNBC cohort although the ethnic distribution has not been documented [1]. The increased expression of AR in the older age group may be in tandem with the physiological rise in androgens relative to oestrogens with increasing age. There was no AR positivity observed in TNBC from patients aged less than 30 years. In the AR positive cohort, the modal age ranged between 50 and 59 years while in the QNBC subjects, the modal age ranged between 30 and 39 years. The modal age range in TNBC with AR positivity and in QNBC in the Safarpour’s study [24] was not statistically different.

In our study, the predominant histologic types were invasive carcinoma, NST which accounted for 75.4%. Other histologic types reported in the current study are invasive metaplastic carcinoma (21.4%), invasive mucinous carcinoma and invasive lobular carcinoma which accounted for 1.6% each. Invasive carcinoma NST (95.5%) accounted for the majority of the histologic phenotype in TNBC while medullary carcinoma, mucinous carcinoma, signet ring cell and papillary carcinoma (4.5%) were one case each in the Zaria study [13]. In the study by Safarpour *et al* [24], the predominant histologic types were poorly differentiated carcinoma, NST and the classic apocrine carcinoma, which accounted for 10 of 18 (55%) TNBC cases studied. Rakha *et al* [33] reported 80.9% of TNBC cases as invasive carcinoma NST and 3.2% were of metaplastic and salivary gland-like carcinoma. The significant number of metaplastic carcinoma also corroborates observations made in an earlier Nigerian study by Titloye *et al* [10]. Invasive carcinoma (NST) constituted the predominant histologic type in all the aforementioned studies [13, 24, 25, 33]. In comparison to Ricciardi *et al* [25], who found a relatively high proportion of lobular carcinoma (15.5%) and medullary carcinoma (6.6%), our study recorded only one lobular carcinoma (1.6%) and no medullary carcinoma. Apart from a small proportion of pleomorphic lobular carcinomas, invasive lobular carcinomas are generally of the luminal phenotype. Histological review and confirmation of the molecular subtype on studying African breast cancer cohorts, as performed in the current study, would therefore be recommended to refine the original histological classification and molecular subtyping.

In the index study, 47.5% of TNBC were intermediate grade while 52.5% were high grade and no low grade tumours were seen. Few of the TNBCs reported by the Zaria study [13] had low grade (6.7%) tumours while majority had either intermediate grade (55.1%) or high grade (38.2%) tumours. This typifies the aggressive biologic behaviour of TNBC cases. Zakaria *et al* [35] reported a tiny proportion of grade 1 carcinoma (9%), with a predominance of grades 2 (58.44%) and grades 3 (32.47%). The tumour grades of TNBC cases observed by Rakha *et al* [33] were 1.77%, 6.73% and 90.78% for grades 1, grades 2 and grades 3, respectively. Most cases reported by Zakaria *et al* [35] and Rakha *et al* [33] were either intermediate grade or high grade. QNBC had relatively higher tumour grade although there was no significant association between AR expression in TNBC cases and tumour grade in our study. Rakha *et al* [33] however observed that the absence of AR expression was associated with higher tumour grade (*p* = 0.001). The lack of significant association between tumour grade and AR expression in our study may be a result, at least partly, because of the small sample size among other things.

Using a threshold of ≥1% for AR expression positivity in line with the ASCO/CAP 2020 guideline applied for ER/PR, 23 of the 61 (37.7%) TNBC cases evaluated were AR positive and 38 cases (62.3%) were AR negative QNBC) [29, 31, 34]. In our study, 23.0% (14 of 23) demonstrate low AR positive expression with 1%–10% nuclei staining proportion while 14.8% (9 of 23) were moderately or strongly positive for AR expression with >10% of nuclei stained. The 2020 updated ASCO /CAP guideline for breast cancer recommended reporting ER positive with 1%–10% of nuclei stained cells as ER low positive category with a recommendation comment, in quote ‘“the cancer in this sample has a low level (1%–10%) of ER expression by IHC’. Though ER low positive category is considered eligible for endocrine therapy, it has also shown heterogeneity in both behaviour and biology. Data from gene expression profile of ER low category have expressed profiles which have been likened to ER negative breast cancer. There are limited data on the overall benefit of endocrine therapies for patients with these results [31].

The Zaria study reported 11.2% of the 89 TNBCs expressed AR positivity [13]. A comparatively high value was reported in Iran where 6/12 (50%) of the TNBC were AR negative [29]. Coincidentally, a similar study of a Japanese TNBC cohort reported that 23 of 61 (37.7%) and 38 of 61 (62.3%) were AR positive and AR negative, respectively [34]. These authors used the same cut off value of 1% for positivity. Ricciardi *et al* [25] investigated 45 Italian TNBC cohorts and reported 26.6% as AR positive and 73.3% QNBC cases. Overall these studies recorded significant number of cases expressing AR (See Tables 4 and 5).

Previous studies have shown variability in the threshold used for reporting AR expression which ranges from ≥1% to ≥10%. Astvatsaturyan *et al* [1] demonstrated and confirmed that ≥1% was the appropriate threshold after using different cut off points. The frequency for AR positive expression was greater when the threshold was changed from 10% to 1%. The frequency of AR positive expression at 10% and 1% threshold is 31.4% (11 of 35) and 40% (14 of 35), respectively. The authors showed no association between AR expression and disease free survival (DFS). The score of ≥1% would also allow larger number of patients to benefit from possible targeted therapies and thus improve their prognosis. Overall the California study cohort, using a threshold of ≥1% AR expression in both the study set and validation set reported 41% (55 of 135) as AR positive [1]. Using the same threshold of ≥1%, another US based study in Yale had a similar result where AR positivity accounted for 36% among 50 TNBC cases. In the Yale cohort, it was observed that changing the minimum threshold for AR positivity from ≥1% to ≥10% would result in categorising 22% of AR positive TNBC cases as AR negative [24]. The Ghanaian study by Proctor *et al* [5], despite having a high incidence of TNBC, reported only 24% as AR positive. Although this proportion is low compared to our study, it still represents a significant proportion of TNBC evaluated. The lower proportion noted in AR expression in the study by Liman *et al* [13] and Proctor *et al* [5] was due to a higher threshold (≥10%) for positive AR expression. Zakaria *et al* [35] in Egypt, also recorded a lower AR positivity (27.27%) compared to our figures. Theirs was an interventional study, whereby patients with AR positive TNBC had 50 mg of the anti-androgen bicalutamide daily. The DFS for 2 and 3 years was 85% and 78%, respectively, while the OS for 2 and 3 years was 100% [35]. Their report serves as a therapeutic indicator of favourable response to clinical trial of a targeted therapy in patients with AR positive TNBC. Antiandrogen therapy has successfully been used for treating metastatic AR positive male breast cancer. Lauro *et al* [38] in 2014 evaluated the activity of cyproterone acetate (antiandrogen) as a monotherapy or in combination with Gonadotropin releasing hormone (GnRH) analogue in metastatic male breast cancer patients. In their study, the overall response was 52.8%. It was concluded that antiandrogens should be considered as a therapeutic continuum for metastatic breast cancer especially if they express AR, although there is no therapy approved for AR TNBC in the international guideline [38].

A limitation of the index study is the lack of follow-up/outcome data for correlation with AR expression. This is a common problem in developing countries where patient care can be fragmented and many patients are lost to follow-up. Addressing this requires a focused multidisciplinary approach, education of patients, better clinical collaboration and cancer registry set-up to improve the quality of follow-up and data collection.

## Conclusion

This study illustrated that TNBC in Nigeria occurred at a young age with a substantial proportion being AR positive which enabled stratification of TNBC into AR positive and AR negative (QNBC), the latter being the predominant group. With emerging and on-going clinical trials using androgen targeted therapy, the need to routinely perform AR IHC for TNBC may become essential in order to identify patients who can benefit from targeted therapy as is being done in other countries. Clinical trials should be set up to investigate whether the improved outcome observed in other climes can be replicated in Nigeria. To clearly demonstrate the response of African women to the available therapeutic agents, a large multicentre study with outcome data will be required to test our hypothesis and provide sufficient evidence to start targeted therapies for AR positive TNBC in Nigeria.

## List of abbreviations

TNBC, Triple negative breast cancer; LAR, Lumina androgen receptor; IHC**,** Immunohistochemistry; TMA, Tissue microarray; AR, Androgen receptor; NST, No special type; QNBC, Quadruple negative breast cancer; ER**,** Estrogen receptor; PR, Progesterone receptor; HER2, Human epidermal growth factor receptor 2; LUTH, Lagos University Teaching Hospital; M, Mesenchymal; OS, Overall survival; HREC, Health Research and Ethics Committee; ASCO/CAP, American Society of Clinical Oncology/College of American Pathologists; ESBR, Elston Ellis Modification of the Scarff Bloom Richardson Grading System; DCIS, Ductal carcinoma *in situ*; DFS, Disease free survival; BRCA1, BReast CAncer gene 1; EDTA, ethylenediaminetetraacetic acid; GnRH, Gonadotropin releasing hormone; UICC, Union for International Cancer Control.

## Conflicts of interest

The author(s) declare that they have no conflicts of interest.

## Funding

This work has been supported by a Union for International Cancer Control (UICC) technical fellowship.

## Figures and Tables

**Figure 4.**
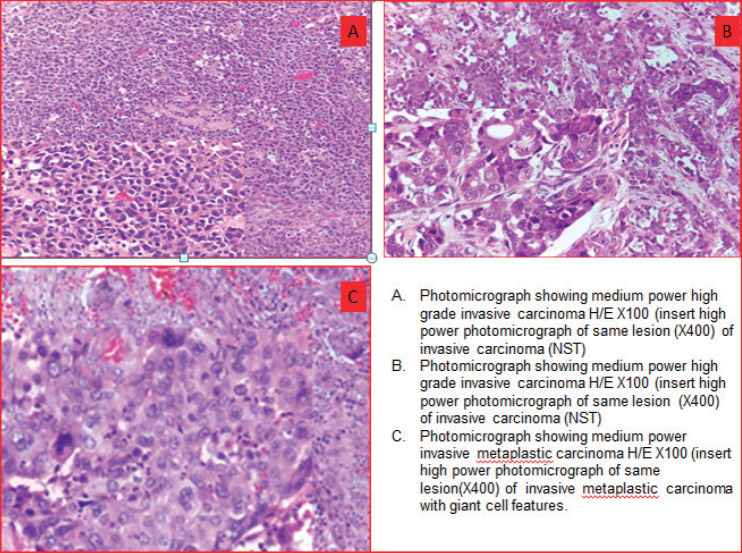
Photomicrographs showing morphologic phenotypes in TNBCs. (a): Medium power high grade invasive carcinoma H/E×100 (insert high power photomicrograph of same lesion (×400) of invasive carcinoma (NST)). (b): Medium power high grade invasive carcinoma H/E×100 (insert high power photomicrograph of same lesion (×400) of invasive carcinoma (NST)). (c): Medium power invasive metaplastic carcinoma H/E×100 (insert high power photomicrograph of same lesion (×400) of invasive metaplastic carcinoma with giant cell features).

**Table 1.** Clinicopathological parameter of the histologic types of TNBC cohort.

Histologic types	Frequency	Percentage
Invasive carcinoma (NST)	46	75.4%
Metaplastic carcinoma	13	21.4%
Mucinous carcinoma	1	1.6%
Lobular carcinoma	1	1.6%
Total	61	100%

**Table 2.** Patient age distribution of selected African studies and Caucasian studies of TNBC.

Study	Age range(years)	Mean/median (years)
Current study, LUTH, Nigeria	24–80	49.3
Liman *et al* [13], Zaria, Nigeria Proctor *et al* [5], Ghana	21–72 28–76	42.89 45
Safarpour *et al* [24], Yale USA Ricciardi *et al* [25], Italy Mohammadizadeh *et al* [29], Iran Rakha *et al* [33], Nottingham, UK Zakaria *et al* [35]	25–93 39–77 - 25–70 19–63	65 58.5 50.94 49.9 35.6

**Table 3.** Clinicopathological parameters of the tumour phenotypes in relationship to AR expression in TNBC.

Invasive carcinoma type	AR positive	AR negative (QNBC)
Invasive carcinoma (NST)	19	27
Metaplastic carcinoma	3	10
Lobular carcinoma	0	1
Mucinous carcinoma Total	1 23	0 38

**Table 4.** Summary of studies of AR expression in TNBC with AR threshold of ≥1%.

Study	No of TNBC cases	Percentages of AR positive TNBC (%)
Current study, LUTH Nigeria	61	37.7%
Astvatsaturyan *et al* [1], California, USA,	135	41.0%
Safarpour *et al* [24], Yale, USA Asano *et al* [34], Japan	50 61	36.0% 37.7%

**Table 5.** Summary of studies of AR expression with threshold of ≥10%.

Study	No. of TNBC cases	Percentage of AR+ TNBC
Proctor *et al* [5], Ghana Liman *et al* [13], Zaria, Nigeria	89 89	24.0% 11.2%
Ricciardi *et al* [25], Italy	45	26.6%
Mohammadizadeh *et al* [29], Iran Zakaria *et al* [35], Egypt	12 77	50.0% 27.3%
